# Expression and Clinical Significance of MDM2 in Non-Functioning PitNETs

**DOI:** 10.3390/medicina59020373

**Published:** 2023-02-15

**Authors:** Xiaohui Yao, Qian Liu, Sida Zhao, Rui Cheng, Chunhui Liu, Gangli Zhang

**Affiliations:** 1 Shanxi Provincial People’s Hospital, Taiyuan 030012, China; 2 Key Laboratory of Central Nervous System Injury Research, Beijing Neurosurgical Institute, Capital Medical University, Beijing 100070, China; 3 Beijing Tiantan Hospital, Capital Medical University, Beijing 100070, China

**Keywords:** non-functioning pituitary neuroendocrine tumors, MDM2, p53, invasion, etoposide

## Abstract

*Background and Objective*: Non-functioning pituitary neuroendocrine tumors (NF-PitNETs) represent a heterogeneous tumor type that lacks effective medical treatment. MDM2, the main negative regulator of p53, binds to and forms a stable complex with p53 to regulate its activity. In this study, we measured the expression levels and role of MDM2 in non-functioning PitNET patients’ combined clinical features and investigated the effect of etoposide on the cell bioactivity of the GT1-1 cell line in vivo and in vitro. *Methods*: RT-PCR and immunochemistry measured the expression levels and role of MDM2 in 103 NF-PitNET patients’ combined clinical features. Cell proliferation, migration, colony and apoptosis experiments measured the effect of etoposide on the GT1-1 cell line in vivo and in vitro. *Results*: There was more invasive behavior (*p* = 0.013) in patients with high MDM2, who were also younger (*p* = 0.007), were more frequently female (*p* = 0.049) and had larger tumor sizes (*p* = 0.018) compared with patients with low MDM2. Patients with high p53 were younger (*p* = 0.017) and had larger tumor sizes (*p* = 0.034) compared with patients with low p53. Univariate (*p* = 0.018) and multivariate (*p* = 0.023) Cox regression analysis showed that MDM2 was the independent factor for invasive behavior in NF-PitNET patients. Log-rank analysis showed that the average progression-free survival (PFS) time in the low MDM2 patients was longer than that in the high MDM2 patients (*p* = 0.044). Functional studies indicated that etoposide inhibited cell proliferation and cell migration and induced apoptosis in p53 independence in GT1-1 cells. Furthermore, etoposide significantly inhibited the growth of GT1-1-xenograft in BALB/c nude mice. The tumor growth inhibition rate of etoposide was 67.4 ± 4.6% after 14 d of treatment, which suggested the anti-tumor activity of etoposide. *Conclusions*: MDM2 played the role of tumorigenesis of NF-PitNET in a p53 independence manner, and an MDM2 inhibitor could be a potential choice for the treatment of NF-PitNET patients.

## 1. Introduction

Non-functioning PitNETs (NF-PitNETs) represent a heterogeneous group of tumors characterized by the lack of endocrine function related to hypersecretion of adenohypophysis hormones [1]. At present, transsphenoidal surgery is the first line treatment for NF-PitNET and is followed by radiotherapy and medical treatment [2]. However, complete tumor removal of invasive or macro NF-PitNETs is still a challenge for neurosurgeons, and overall remission prevalence was 47.6% (95% CI = 40.8–54.4%) in invasive macroadenomas patients and 76.4% (95% CI = 72.2–80.1%) in macroadenomas patients. Residual as well as recurrent tumors are frequent and this affects the overall prognosis [3]. To date, there is no guide or expert consensus on the medical treatment of NF-PitNETs; although, there are several clinical trials such as those for agonists of dopamine receptor type 2 (DRD2) [4] and somatostatin receptor type 2 (SSTR2) [5].

As a tumor suppressor, p53 is a crucial regulator of cellular homeostasis linked to cancer development, which represents a main molecular target of genetic diagnostics and therapeutic interventions [6]. Usually, the activity of p53 is low due to the interacting protein, mouse double minute 2 homolog (MDM2), which has E3 ubiquitin ligase activity that degrades p53 via the proteasome system [7]. Overexpression of MDM2 could modulate various proangiogenic pathways and induce tumor angiogenesis in several tumors, including gastric cancer, breast carcinoma, prostate cancer and ovarian carcinoma [8,9]. MDM2 promoted cell proliferation and inhibited apoptosis in the pituitary adenoma cell line AtT-20 by directly interacting with p53 [10]. We previously reported that there were more MDM2-positive samples in recurrence patients than in primary patients among 35 paired recurrence/regrowth NF-PitNET patients [11]. Activation of p53 by inhibitors of the p53-MDM2 interaction is being pursued as a therapeutic strategy in p53 wild-type cancers [12]. Phase I trial of an MDM2 inhibitor in advanced solid tumors showed an acceptable safety, tolerability and efficacy [13,14,15]. 

Although large placebo-controlled clinical trials are lacking, there were effective treatments for NF-PitNETs after incomplete, including temozolomide [16], combination treatment with somatostatin receptor ligands plus dopamine agonists [1]. In this study, we measured the expression levels and role of MDM2 in 103 NF-PitNET patients’ combined clinical features and investigated the potential role of etoposide, an MDM2 inhibitor, in the prevention of NF-PitNETs in vivo and in vitro. 

## 2. Materials and Methods

### 2.1. Patients, Tissue Specimens and Cell Lines

We retrospectively reviewed 103 NF-PitNET patients in Beijing Tiantan Hospital from July 2012 to December 2016. The patients underwent plain and enhanced head MRI, thin layer skull base CT scanning and three-dimensional reconstruction. The samples were obtained using an immunohistochemical and transmission electron microscope. The diagnosis of NF-PitNETs was based on the pituitary transcription factor and hormone immunohistochemical staining according to the fourth edition of the WHO classification. The study was a retrospective study and the protocols were approved by the Internal Review Board (IRB) of Beijing Tiantan Hospital affiliated with Capital Medical University and were conducted according to the principles expressed in the Declaration of Helsinki.

A mouse GT1-1 cell line was cultured in Dulbecco’s modified Eagle’s medium (DMEM; Gibco-BRL, Gaithersburg, MD, USA) supplemented with 2.5% fetal bovine serum + 10% horse serum in a humidified incubator at 37 °C in 5% CO_2_. The culture medium was replaced every other day.

### 2.2. Reverse Transcription and Quantitative PCR

Microarray hybridization and qRT-PCR were performed as previously described [17]. The total RNA of 30 samples was extracted and purified using the Rneasy^®^Mini Kit (QiaGen, Hilden, Germany) following the manufacturer’s instructions. RT-qPCR was performed on a QuantStudio5 (Applied biosystems, Singapore). The fold-change in the differential expression for each gene was calculated using the comparative CT method (2^−∆∆CT^ method) in an R package with “PCR” functions (https://github.com/MahShaaban/pcr, accessed on 2 January 2023), a GAPDH reference gene and the “1/2a” stage reference group [18]. The primers of genes are listed in Supplementary Table S1.

### 2.3. TMA Construction and Immunohistochemistry (IHC)

Three 2.0 mm diameter core biopsies were selected from the paraffin-embedded tissue blocks. The cores were transferred to tissue microarray (TMA) and the pathologists were blinded to the identity of the TMA slides as previously described [19]. IHC tests with a rabbit monoclonal anti-Ki-67 antibody (1:1000, LEICA), an anti-MDM2 antibody (1:300, Abcam), and an anti-p53 (1:400, Abcam) antibody were performed using the Leica Bond-III system (Aperio, CA, USA). The expression of slides was examined by an Aperio AT2 digital scanner (Leica Biosystems). The H-score was obtained by multiplying the staining intensity by a constant to adjust the mean to the strongest intensity [H-score = 3 × (percentage of strong staining)] (1.0%, weak; 2.0%, moderate; 3.0%, strong) to give a score ranging from 0 to 300. 

### 2.4. Cell Proliferation, Cell Colony Formation Assay, Cell Migration and Invasion Assays

The GT1-1 cells were adjusted to a density of 1 × 10^5^ cells/mL. A volume of 100 µL of cell suspension was added to each well of a 96-well plate and given different concentrations of etoposide (20 nM and 100 nM, E1383, Sigma) for 24 h, 48 h and 72 h. Then, 20 µL of 3-(4,5-diethylthiazol-2-yl) -5-(3-carboxymethoxyphenyl)-2-(4-sulfophenyl)-2H-tetrazolium inner salt (MTS) solution was added to each well and further incubated for 4 h. The absorbance at 490 nm of each well was measured using an ELISA plate reader (Thermo Fisher, Waltham, MA, USA).

For the colony formation assay, a total of 200 cells were plated into each well of a 12-well plate and maintained for 14 days. The colonies were then fixed with 4% formaldehyde for 30 min and stained with 0.2% crystal violet for 10 min. Triplicate wells were measured in each treatment group.

The migration and invasion of GT1-1 cells were measured using modified Transwell chambers (Corning, Corning, NY, USA) coated with fibronectin and Matrigel in 24-well culture plates (BD Biosciences, San Jose, CA, USA). The GT1-1 cells (5 × 10^5^ cells) with different concentrations of etoposide (20 nM and 100 nM) were added onto membranes coated with fibronectin and Matrigel. After 24 h, migratory cells adhering to the lower membrane were fixed in 4% paraformaldehyde and stained using Harris stain. The average number of migratory cells was quantified by counting five random high-powered fields (100×) under a microscope (ZEISS, Jena, Germany). All experiments were performed in triplicate. 

### 2.5. SDS–PAGE and Western Blot Analyses

The GT1-1 cells were lysed in TNE buffer (50 mM Tris–HCl, pH 7.4, 150 mM NaCl, 1 mM EDTA; all from Sigma-Aldrich, St. Louis, MO, USA) containing 1% Nonidet P-40 (Calbiochem, San Diego, CA, USA) with protease and phosphatase inhibitor cocktails (Roche). The total extracts were centrifuged at 12,000 rpm for 30 min at 4 °C, and the protein concentration was determined with the BCA method (Pierce Biotechnology, Waltham, MA, USA). For Western blot analysis, 40 µg of lysate per lane was loaded onto 10% Bis-Tris SDS-PAGE gels and blotted onto polyvinylidene fluoride (PVDF) membranes. Different blots were incubated with antibodies against anti-Bcl-xl2 (1:1000, Abcam), anti-Bax (1:1000, Abcam), anti-E-CAD (1:8000, Abcam), anti-MDM2 (1:1000, Abcam), anti-p-MDM2 (1:1500, Abcam), anti-MMP2 (1:2000, Abcam), anti-N-CAD (1:10,000, Abcam), anti-p21 (1:2000, Abcam), anti-p53 (1:1000, Abcam), anti-vimentin (1:2000, Abcam), anti-β-actin (1:8000, Sigma) and GAPDH (1:10,000, Sigma) followed by secondary antibodies tagged with horseradish peroxidase (Santa Cruz Biotechnology). Blots were visualized by enhanced chemiluminescence, and densitometry was performed with an Amersham Imager 600. GAPDH levels were analyzed as a loading control.

### 2.6. TUNEL Staining for Apoptosis Assay

A TUNEL staining kit (Roche, Swiss) was used to assess cell apoptosis. The TUNEL assay was performed in triplicate with independent tumors from each treatment group. The percentage of apoptosis was calculated by dividing the number of TUNEL-positive cells by the total cells.

### 2.7. Mouse Xenograft Model

Animal experiments were performed using 6-week-old male BALB/c nude mice (SCXK2012-0001). Groups of five animals each were housed in an animal room at 23 ± 2 °C, 55 ± 5% humidity and a 12 h light, 12 h dark cycle. The mice were fed a standard, unrestricted diet. The GT1-1 cells were harvested, resuspended in PBS at 1 × 10^7^ cells/mL and 200 µL of the cell suspension was injected into the flanks of mice on day 0. Intraperitoneal injection of 20 mg/kg body weight etoposide (20 mg/kg group) or PBS (vehicle group) was administered daily on day 21. Tumors were measured with calipers, and the volumes were calculated as (3.14 × length × width × depth)/6 on days 0, 3, 7 and 14. After 15 days, the mice were euthanized and the tumors were removed. All animal experiments were approved by the Animal Care and Use Committee of Beijing Neurosurgical Institute.

### 2.8. Statistic

χ^2^ exact tests and Pearson’s test were used to determine the significance of clinicopathologic characteristics variables. A t-test was applied to the examination of differential levels of MDM2 and p53 in patients. A one-way ANOVA test and a subsequent t-test were used in cell functional experiments. All *p* values are two-sided and 0.05 was applied as the significance level.

## 3. Results

### 3.1. Clinical Features of 103 NF-PitNET Patients

The clinical features of the 103 patients included are shown in Supplementary Table S2. Patient ages were from 21 to 75 years (mean, 51.67 ± 1.05 years). Tumor diameters ranged from 1.3 to 5.2 cm (median size: 3.4 cm). Of the 103 cases, 70 were male and 33 were female. The diagnosis of invasive pituitary adenomas was adopted by 1) Knosp classification grade III–IV tumors or Hardy classification III–IV, 2) Ki-67 > 3%. A total of 44/103 patients were invasive adenomas including 24 males and 20 females. During the follow-up, the recurrence cases were 19/103 (18.4%). 

### 3.2. MDM2 and p53 Expression Profiles and Correlations with Clinicopathologic Parameters in NF-PitNET Patients

In this study, RT-PCR and IHC were used to evaluate the expression levels of MDM2 and p53 in these patients. The 103 patients were divided into a high group (n = 51) and a low group (n = 52) according to the median of the MDM2 (mean 0.062 ± 0.004, cutoff value 0.055) or p53 (mean 0.127 ± 0.012, cutoff value 0.105) mRNA levels in Table 1.

There was a lower age (48.8 ± 1.63 vs. 54.5 ± 1.24 years, *p* = 0.007), more females (21/51 vs. 12/52, *p* = 0.049), larger tumor sizes (16.79 ± 4.03 vs. 6.63 ± 1.15 cm^3^, *p* = 0.018) and more invasive behavior (28/51 vs.16/52, *p* = 0.013) in high MDM2 patients than in low MDM2 patients. We also noticed a lower age (49.14 ± 1.64 vs. 54.15 ± 1.26 years, *p* = 0.017) and larger tumor sizes (16.29 ± 4.08 vs. 7.14 ± 1.04 cm^3^, *p* = 0.034) in high p53 patients than in low p53 patients, and no statistic difference in gender and invasive behavior (*p* > 0.05). Univariate Cox regression analysis showed female patients, recurrence and patients with high MDM2 levels had more chance of invasive behavior (all *p* < 0.05). Univariate and multivariate Cox regression analysis both showed that MDM2 and recurrence were the independent factors for invasive behavior in NF-PitNET patients in Table 2. 

**Figure 1:**
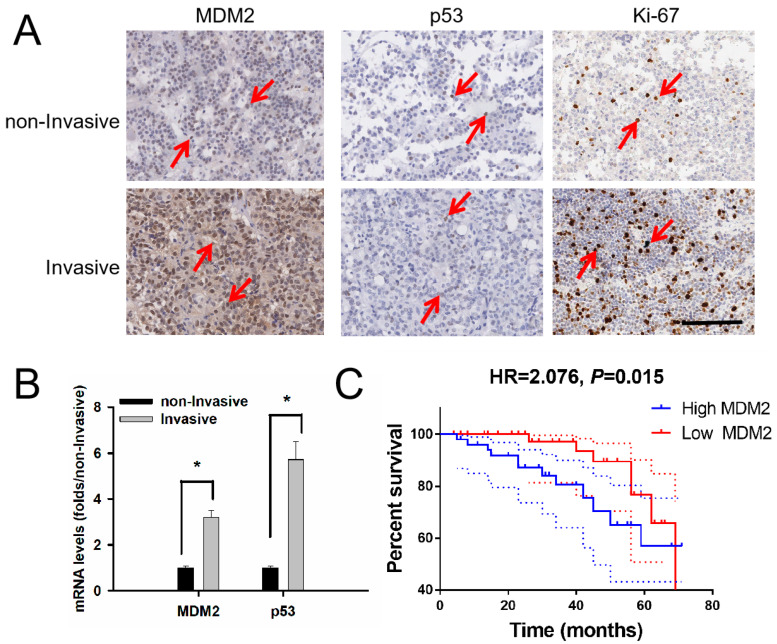
Levels of MDM2 related to tumor invasive behavior and recurrence time in 103 NF-PitNET patients. (**A**) Immunohistochemistry image of MDM2, p53 and Ki-67 in PitNET patients. Red arrow: positive cell, 200×. (**B**) The mRNA levels of MDM2 and p53 in invasive patients were higher than those in non-invasive patients. * compared to the non-invasive group, *p* < 0.05. (**C**) Patients with high MDM2 had a shorter PFS time compared with patients with low MDM2. Red: low MDM2 group, blue: high MDM2 group, HR = 2.076, *p* = 0.015.

According to the H-score of staining in the IHC experiment, there was a statistical difference in MDM2 (166.7 ± 4.73 vs. 129.7 ± 5.04, t = 5.168, *p* < 0.001) and p53 levels (81.56 ± 13.01 vs.57.5 ± 5.05, *p* = 0.047) in the invasive samples compared with those in the non-invasive samples in Figure 1A. The RT-PCR results showed that the mRNA level of MDM2 in invasive patients was 3.19 ± 0.32 that of non-invasive patients (t = 2.461, *p* = 0.016). The mRNA level of p53 in invasive patients was 5.73 ± 0.78 that of non-invasive patients (t = 2.066, *p* = 0.041) in Figure 1B. Log-rank analysis showed that patients with low MDM2 expression had significantly longer disease-free survival than those with high MDM2 expression in Figure 1C (*p* = 0.015). 

### 3.3. Evaluation of the MDM2 Inhibitors Etoposide on Cell Bioactivity of GT1-1 Cells

**Figure 2:**
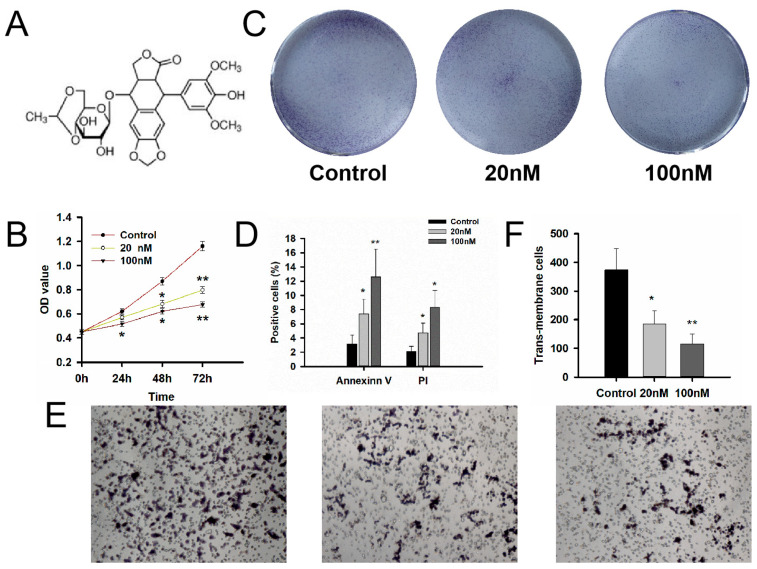
Etoposide, an MDM2 inhibitor, inhibited the bioactivity of GT1-1 cells. (**A**) The structure of the MDM2 inhibitor, etoposide. (**B**) Etoposide could inhibit the cell viability after 24 h, 48 h and 72 h treatment in a dose and time manner. (**C**) Etoposide reduced the colony of GT1-1 cells. (**D**) Annexin V and PI experiments showed that etoposide induced the apoptosis of GT1-1 cells. (**E**) The transwell experiment showed that etoposide reduced the transmembrane cells after 24 h treatment. Magnification ×50. (**F**) Statistical data of the Transwell experiment. n = 3. * compared with the control group *p* < 0.05, ** *p* < 0.01.

In order to evaluate the effect of etoposide, we tested the cell viability, apoptosis and migration ability with different concentrations of drugs in NF-PitNET GT1-1 cell lines. The structure of etoposide was shown in Figure 2A. MTS results showed that cell viability in the 20 nM etoposide group was 92.4%, 78.3% and 68.7% after 24 h, 48 h and 72 h treatment, and 83.2%, 71.3% and 58.4% in the 100 nM group in Figure 2B. Clone forming experiments also suggested that the etoposide inhibited cell proliferation in the GT1-1 cell line in Figure 2C. Flow cytometry showed Annexin V positive cells of the etoposide group were 7.4 ± 2.1% and 12.6 ± 3.9 after 24 h treatment compared to 3.2 ± 1.2% in the control group. The PI positive cells of the etoposide group were 4.7 ± 1.4% and 8.3 ± 2.9% compared to 2.1 ± 0.7 in the control group in Figure 2D. A Transwell experiment measured the cell invasion and migration of GT1-1 cells after etoposide treatment. The transmembrane cells were reduced to 184 ± 47 and 116 ± 34 after etoposide treatment, and 373 ± 74 in the control group (*p* < 0.05) in Figure 2E,F. 

### 3.4. MDM2 Inhibitor Etoposide Regulated p53 Stability and Related to Epithelial Mesenchymal Transition (EMT) in GT1-1 Cell Line

**Figure 3:**
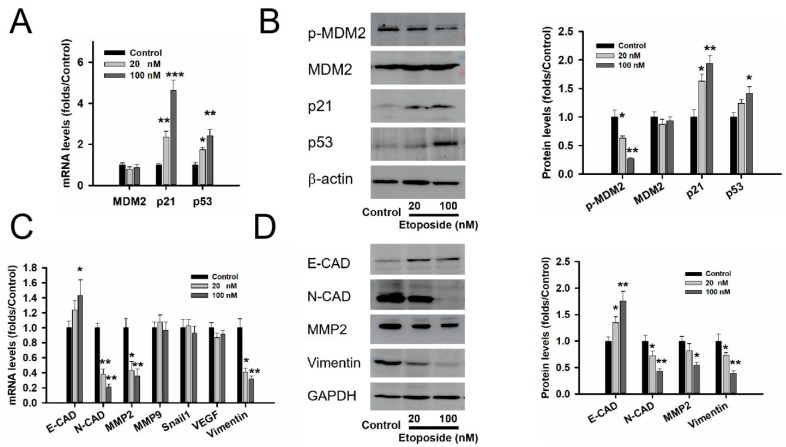
Etoposide induced the expression of p21 and inhibited the genes related to EMT in GT1-1 cells. (**A**) The RT-PCR experiment showed that etoposide upregulated the mRNA levels of p21 and p53 in GT1-1 cells. (**B**) The Western blot experiment showed that etoposide relieved the phosphorylation level of MDM2 and upregulated the level of p21 in a dose manner. RT-PCR (**C**) and Western blot (**D**) experiments showed that etoposide upregulated the levels of E-CAD and mainly inhibited the levels of MMP2, N-CAD and vimentin in GT1-1 cells. n = 3. * compared with the control group *p* < 0.05, ** *p* < 0.01, *** *p* < 0.001.

RT-PCR and Western blot experiments showed that etoposide relieved the phosphorylation level of MDM2, not the total protein of MDM2 in Figure 3A,B. Next, we explored the connection between etoposide and p53. We found the activated p53 expression gradually increased with the dose of etoposide despite the mRNA level in Figure 3A or protein level in Figure 3B (*p* < 0.05). We found that the mRNA and protein levels of p21 accumulated with the dose of etoposide in the GT1-1 cells (*p* < 0.05). 

Since EMT plays a crucial role in the early steps of metastasis, we further detected whether MDM2 was associated with the EMT of GT1-1 cells. The RT-PCR experiment measured the mRNA levels of genes related to EMT. The level of N-CAD, MMP2 and vimentin were impaired with the dose of etoposide, and the level of E-CAD was heightened in Figure 3C (*p* < 0.05). A Western blot experiment also showed the same tendency in Figure 3D (*p* < 0.05).

### 3.5. MDM2 Inhibitor Etoposide Showed the Anti-Proliferative Role in GT1-1 Xenograft Mouse Model

**Figure 4:**
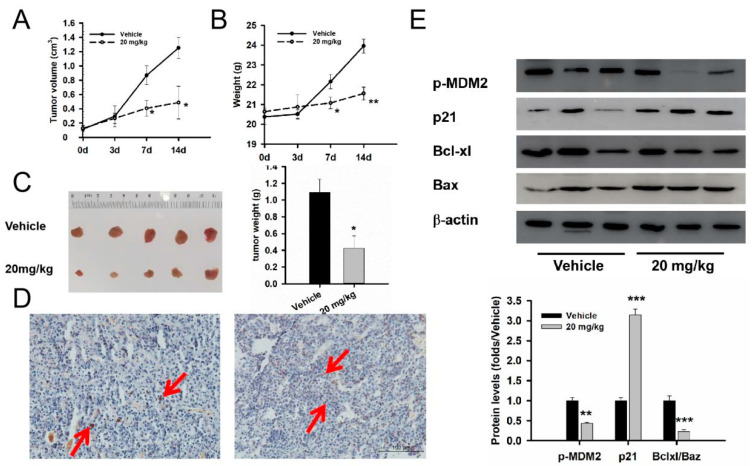
Etoposide played the anti-tumor effect in GT1-1 cell xenograft mice. (**A**) Tumor volume changes after etoposide treatment from day 0 to day 14. (**B**) Mice weight changes after etoposide treatment from day 0 to day 14. (**C**) Tumor weight changes after etoposide treatment. The average weight in the vehicle group was 1.09 ± 0.16 g, and 0.42 ± 0.15 g in the etoposide group (t = 3.065, *p* = 0.016). (**D**) TUNEL staining showed more TUNEL-positive cells in the etoposide group than in the vehicle group, as the red arrow shows. Bar: 100 μm. (**E**) Western blot experiment showed that etoposide relieved the phosphorylation level of MDM2 and the ratio of Bcl-xl/Bax, and upregulated the protein of p21. n = 3–5. * compared with the control group *p* < 0.05, ** *p* < 0.01, *** *p* < 0.001.

In the GT1-1 xenograft, the mouse weight and tumor volume of the vehicle group gradually increased over time in Figure 4A,B. The tumor growth inhibition values were 53.1 ± 20.2% on day 7 (t = 2.629, *p* = 0.039) and 61.1 ± 18.1% on day 14 (t = 3.77, *p* = 0.015) after etoposide the treatment in Figure 4B. The average tumor weight of the 20 mg/kg group was 38.9 ± 13.7% of the vehicle group (t = 3.065, *p* = 0.016) in Figure 4C. The TUNET staining experiment showed more apoptosis cells in the 20 mg/kg group compared with that in the vehicle group, as the red arrow shows in Figure 4D. The Western blot experiment showed that etoposide inhibited the phosphorylation of MDM2 and lowered the ratio of Bcl-xl/Bax in xenograft tumor samples, as shown in Figure 4E. 

## 4. Discussion

The disorder of the cell cycle plays an important role in the pathogenesis of non-functioning PitNETs, and the critical roles of proteins involved in cell cycle arrest, control and the response to DNA damage are unclear [20]. Cell cycle arrest could relieve the excessive proliferative response to keep the pituitary homeostasis. p53/MDM2 play the roles in cell-cycle arrest at the G1/G2 checkpoints and apoptosis [21]. Cell-cycle arrest in the G1 phase is often mediated by p21 [22]. In this study, we focused on the expression levels and role of MDM2 in 103 NF-PitNET patients’ combined clinical features and investigated the MDM2 inhibitor, etoposide, with regards to the cell proliferation and invasion of the GT1-1 cell line and GT1-1-xenograft model. The patients with high MDM2 were younger and had more invasive behavior, larger tumor volumes and shorter recurrence times compared with patients with low MDM2. In vivo and in intro experiments both showed that the MDM2 inhibitor etoposide relieved the tumor proliferation and migration of NF-PitNETs by inhibiting the activity of MDM2. 

MDM2 was discovered by Olineret et al. in 1992, which was located on the 12q13-14 locus and encodes a 483-amino acid protein [23]. MDM2 amplification has been detected in many human malignancies, including lung cancer, colon cancer and other malignancies, and the phenomena of amplification occurs more frequently in metastatic and recurrent cancers compared to non-malignant tumors [24]. In a large cohort of PitNETs, there was a tendency that the p53 indexes were higher in recurrent corticotroph adenomas and lactotroph adenomas but the values did not reach a significant level [25]. In our past study, there were more MDM2-positive cases in recurrence patients in 35 pair primary and recurrence/regrowth patients. In this study, we noticed that there was a shorter PSF time in patients with high MDM2 compared to patients with low MDM2, although there was no statistical difference in the recurrence rate between the two groups. However, univariate and multivariate Cox regression both showed that MDM2 was the independent factor of invasive behavior and not recurrence in NF-PitNETs. In other words, there was still a lack of MDM2 with clinical outcomes except for clinical behavior (age, gender and invasive behavior). 

Our data showed that MDM2 triggered tumorigenesis and increased the metastatic ability through the modulation of various genes related to migration and invasion. Etoposide, an inhibitor of MDM2, is clinically used for the treatment of several cancers by inhibiting topoisomerase II, an enzyme responsible for DNA strand ligation during cell division [26]. In vitro experiments showed that etoposide inhibited cell proliferation and migration in the GT1-1 cell line. RT-PCR and Western blot experiments both showed that etoposide relieved the phosphorylation of MDM2, not total protein. N-CAD and vimentin were downstream molecular targets regulated by the activity of MDM2. Our data were in accord with the silencing of MDM2 significantly inhibiting the expression of EMT-related genes N-cadherin and vimentin in PC9 cells [27]. In vivo experiments showed etoposide had a strong tumor growth inhibition value in GT1-1- xenograft mice. TUNEL staining showed etoposide-induced apoptosis in vivo, and the upward Bcl-xl/Bax ratio proved the activation of apoptotic signaling in xenograft samples. 

The normal process of cell division occurs via the cell cycle, and the ability to sustain unscheduled proliferation is a hallmark of cancer. The medical treatment of targeting the cell cycle has long been appreciated but the translation of this approach to the bedside was initially limited by the low specificity of early cell-cycle inhibitors [28]. In tumor growth regulation and metastatic process, oncogene MDM2 showed a regulatory role in cell cycle control differentiation, DNA repair, gene transcription and cell fate in a p53-dependent or independent manner [29]. As a factor of atypical adenomas in the 2004 WHO classification, p53 nuclear staining suggested that p53 maybe be an oncogene, not a tumor suppressor gene. In a prospective study, p53 immunoreactivity appeared to positively correlate with tumor invasive behavior (*p* = 0.003) [30]. In this study, RT-PCR and IHC experiment results did not see the opposite trend between the levels of MDM2 and p53 in NF-PitNET patients. Based on these findings, we speculated that MDM2 played the oncogene role in a p53-independent manner in NF-PitNETs. We found that the change in p21 was more significant compared with p53 after the etoposide treatment in vitro experiment. It was recognized that initiation of the G1 cell cycle checkpoint involves rapid degradation of cyclin D1 but, to maintain the checkpoint, p21 is required [31]. In fact, p21 was uniquely involved in maintaining the G1 cell cycle arrest when the checkpoint was triggered.

## 5. Conclusions

In summary, MDM2 might be associated with tumor proliferation and invasive behavior in NF-PitNETs. In vivo and in vitro experiments suggested that MDM2 played the oncogene role in a p53-independent manner, and the MDM2 inhibitor etoposide could inhibit the proliferation and migration in GT1-1 cells and induce the apoptosis. 

## Figures and Tables

**Table 1:** Association of MDM2 and p53 expression with clinico-pathological characteristics.

Variable	MDM2		*p* Value	p53		*p* Value
	High (n = 51)	Low (n = 52)		High (n = 51)	Low (n = 52)	
Gender			0.012			0.012
Male	32	38		46	24	
Female	19	14		13	20	
Age	48.8 ± 1.63	54.5 ± 1.24	0.007	53.01 ± 1.18	49.39 ± 1.86	0.088
Tumor size (cm^3^)	16.79	6.63 ± 1.15	0.018	6.86 ± 1.13	18.53 ± 4.75	0.008
Recurrence			0.012			0.012
Yes	6	13		6	13	
No	53	31		53	31	
Invasive behavior						
Yes	30	14	0.001	24		
No	21	38		27		
Cavernous sinus compression			0.061			0
Yes	17	9		5	21	
no	34	43		54	23	

**Table 2:** Univariate and multivariate Cox regression analysis of the invasive behavior of 103 PitNET patients.

Kinds	Univariate Cox				Multivariate Cox			
	*p* Value	HR	95%CI Down	95%CI Up	*p* Value	HR	95%CI Down	95%CI Up
MDM2	0.018	1.048	1.008	1.090	0.023	1.151	1.019	1.299
TP53	0.066	1.100	0.994	1.217	0.281	1.079	0.940	1.240
Age	0.075	0.966	0.930	1.004	0.280	0.977	0.936	1.019
Gender	0.039	0.409	0.176	0.954	0.091	0.454	0.181	1.135
Recurrence	0.016	3.704	1.278	10.735	0.043	3.296	1.037	10.477
Tumor size	0.119	1.020	0.995	1.046	0.239	1.014	0.991	1.038

## Data Availability

All the data generated or analyzed in this study are included in this published article and its additional files. Details can be obtained from the corresponding author.

## Supplementary Materials

The following supporting information can be downloaded at: https://www.mdpi.com/article/10.3390/medicina59020373/s1, Table S1: Primers in RT-PCR experiment; Table S2: Clinic-pathological features of 103 PitNETs patients.

## Abbreviations

Dopamine receptor type 2, DRD2; epithelial mesenchymal transition, EMT; immunohistochemistry, IHC; mouse double minute 2 homolog, MDM2; non-functioning pituitary neuroendocrine tumors, NF-PitNETs; tissue microarray, TMA.
