# Supplementary material for: Expression and Clinical Significance of MDM2 in Non-Functioning PitNETs
Source: Medicina (Kaunas). 2023 Feb 15;59(2):373. doi: 10.3390/medicina59020373 (PMC9963423; doi:10.3390/medicina59020373)
Supplement: Supplementary file 1 [file medicina-59-00373-s001.zip › medicina-2172757-supplementary.pdf]

Supplementary Table S1 Primers in RT-PCR experiment

Gene	Up:	Down:
hMDM2	GTGTTCAGTGGCGATTGGAG	TAACCAGGGTCTCTTGCTCC
mMDM2	AGTTCTCACGAAGGGTCCAG	TGGAAGTCGATGGTTGGGAA
hTP53	aggttggctctgactgtacc	CACCTCAAAGCTGTTCCGTC
mhTP53	GTACCGTATGAGCCACCTGA	CACGAACCTCAAAGCTGTCC
mE-CAD	CAACCCAAGCACGTATCAGG	TGACAACAACGAACTGCTGG
mN-CAD	TGTGGAGGCTTCTGGTGAAA	CCTTGAAATCTGCTGGCTCG
mMMP2	GAACACCATCGAGACCATGC	ATCATCCACGGTTTCAGGGT
mMMP9	AAAGGCCATTCGAACACCAC	GGATGACAATGTCCGCTTCG
mSNAIL	AGCCTCCTACCCCTCAGTAT	GGGGAGGGGAACTATTGCAT
mVEGF	CTTTCTGCTCTCTTGGGTGC	CACAGGACGGCTTGAAGATG
mVimentin	TCTGTGTCCTCGTCCTCCTA	CGAGAAGTCCACCGAGTCTT
hGAPDH	CACATCGCTCAGACACCATG	TGACGGTGCCATGGAATTTG
mGAPDH	GGAGAAAGTGGGGAAAAGCC	GAACAGGGAGGAGCAGAGAG

Supplementary Table S2. Clinic-pathological features of 103 PitNETs patients

**Variable**	Invasive behavior		*P* value
	No (n=59)	Yes (n=44)	
**Gender**			0.012
male	46	24	
female	13	20	
Age	53.01±1.18	49.39±1.86	0.088
Tumor size (cm3)	6.86±1.13	18.53±4.75	0.008
Recurrence			0.012
yes	6	13	
no	53	31	
Cavernous sinus compression			0
yes	5	21	
no	54	23	
